# Tobacco Upregulates *P. gingivalis* Fimbrial Proteins Which Induce TLR2 Hyposensitivity

**DOI:** 10.1371/journal.pone.0009323

**Published:** 2010-05-04

**Authors:** Juhi Bagaitkar, Donald R. Demuth, Carlo Amorin Daep, Diane E. Renaud, Deanne L. Pierce, David A. Scott

**Affiliations:** 1 Department of Microbiology and Immunology, School of Medicine, University of Louisville, Louisville, Kentucky, United States of America; 2 Oral Health and Systemic Disease Research Group, Department of Oral Health and Rehabilitation, School of Dentistry, University of Louisville, Louisville, Kentucky, United States of America; Columbia University, United States of America

## Abstract

**Background:**

Tobacco smokers are more susceptible to periodontitis than non-smokers but exhibit reduced signs of clinical inflammation. The underlying mechanisms are unknown. We have previously shown that cigarette smoke extract (CSE) represents an environmental stress to which *P. gingivalis* adapts by altering the expression of several virulence factors – including major and minor fimbrial antigens (FimA and Mfa1, respectively) and capsule – concomitant with a reduced pro-inflammatory potential of intact *P. gingivalis*.

**Methodology/Principal Findings:**

We hypothesized that CSE-regulation of capsule and fimbrial genes is reflected at the ultrastructural and functional levels, alters the nature of host-pathogen interactions, and contributes to the reduced pro- inflammatory potential of smoke exposed *P. gingivalis*. CSE induced ultrastructural alterations were determined by electron microscopy, confirmed by Western blot and physiological consequences studied in open-flow biofilms. Inflammatory profiling of specific CSE-dysregulated proteins, rFimA and rMfa1, was determined by quantifying cytokine induction in primary human innate and OBA-9 cells. CSE up-regulates *P. gingivalis* FimA at the protein level, suppresses the production of capsular polysaccharides at the ultrastructural level, and creates conditions that promote biofilm formation. We further show that while FimA is recognized by TLR2/6, it has only minimal inflammatory activity in several cell types. Furthermore, FimA stimulation chronically abrogates the pro-inflammatory response to subsequent TLR2 stimulation by other TLR-2-specific agonists (Pam3CSK4, FSL, Mfa1) in an IκBα- and IRAK-1-dependent manner.

**Conclusions/Significance:**

These studies provide some of the first information to explain, mechanistically, how tobacco smoke changes the *P. gingivalis* phenotype in a manner likely to promote *P. gingivalis* colonization and infection while simultaneously reducing the host response to this major mucosal pathogen.

## Introduction

Tobacco smokers are more susceptible than non-smokers to multiple infectious diseases, particularly mucosal infections such as tuberculosis, pneumonia, Chlamydiasis, gonorrhoea, otitis media and periodontitis [1]. The mechanisms underlying such increased susceptibility are not well understood. Several groups have shown that tobacco smoke as well as individual smoke components induce physiological and structural changes, e.g. reduction of mucociliary clearance [2], [3], and dysregulate specific elements of immune function, e.g. inhibition of the respiratory burst and phagocytosis [4], [5], [6] and interference in antigen presentation [7], [8]. However, the influence of tobacco on bacterial virulence is, essentially, unstudied.

Periodontitis is a bacteria-induced, irreversible chronic inflammatory mucosal disease characterized by the destruction of the soft and hard supporting structures of the teeth. Tobacco smokers are more susceptible than non-smokers to infections with periodontal pathogens [1], are more likely to develop severe periodontitis and to prove refractory to treatment [9]. Paradoxically, smokers show reduced clinical signs of inflammation in response to dental plaque than non-smokers, particularly the key diagnostic indices of gingival bleeding on probing and edema [9], [10]. Again, the mechanisms underlying this phenomenon are poorly characterized.

*Porphyromonas gingivalis*, a Gram negative, asaccharolytic anaerobe, is a key periodontal pathogen whose numbers are increased in tobacco smokers [9], [10]. There is some evidence that components of tobacco smoke augment *P. gingivalis* pathogenesis. Nicotine and its primary metabolite, cotinine, have been shown to increase the lethality of cell-free extracellular toxins and cell lysates from *P. gingivalis* in the chick embryo model [11], [12]. The combination of benzopyrene, a tobacco smoke aryl hydrocarbon, and *P. gingivalis* lipopolysaccharide (LPS) significantly increase the inhibition of osteogenesis in a rat bone marrow cell model compared to either agonist alone [13].

We have recently shown that *P. gingivalis* adapts to the environmental stress presented by cigarette smoke extract (CSE) by altering the expression of several genes and outer membrane proteins [14]. Concomitant with this adaptive response to CSE, *P. gingivalis* induces a lower inflammatory response (TNF-α, IL-6 and IL-12 p40) from human innate cells compared to unexposed, control bacteria [14]. Furthermore, the inflammation-inducing potential of *P. gingivalis* is restored when cells are sub-cultured back into fresh medium without CSE. Interestingly, microarray analyses determined that specific genes (PG2133 and PG2134) in operons coding for the synthesis and assembly of major and minor fimbrial antigens (FimA and Mfa1) of *P. gingivalis* were induced on exposure to CSE, while several genes in the capsular biosynthesis locus (*capK*, PG0117, PG0118 and *wecC*) were suppressed [14].

It is important to note that capsule polysaccharides and major fimbrial protein will be the two *P. gingivalis* features that first engage the host response and, thus, are likely to play critical roles in directing initial host-pathogen interactions. Capsule production is associated with tissue invasiveness [15] and has been reported to be inversely related to biofilm growth [16], while capsular polysaccharides represent potent cytokine-inducing stimuli [17]. The major fimbrial antigen, FimA, is also an important virulence factor that facilitates the adhesion and initial attachment of *P. gingivalis* to junctional epithelial cells, thus aiding sub-gingival colonization [18]. FimA appears to signal via TLR2 and induces the expression of several pro-inflammatory cytokines such as TNF-α, IL-6 and IL-1β in innate immune cells [19]. However, the potency of FimA as a pro-inflammatory agonist is controversial [20], [21], [22], [23]. Thus, alterations in *P. gingivalis* capsule and fimbriae production would be expected to exert marked effects on virulence and host-pathogen interaction.

We hypothesized that CSE-regulation of capsule and fimbrial genes is reflected at the ultrastructural and functional levels, alters the nature of host-pathogen interactions, and contributes to the reduced pro- inflammatory potential of smoke exposed *P. gingivalis*. We establish that CSE up-regulates *P. gingivalis* FimA at the protein level, suppresses the production of capsular polysaccharides at the ultrastructural level, and creates conditions that promote biofilm formation. We further show that while FimA is recognized by TLR2/6, it has only minimal inflammatory activity in several cell types (PBMCs, neutrophils, and epithelial cells) and, furthermore, FimA stimulation chronically abrogates the pro-inflammatory response to subsequent TLR2 stimulation by other TLR2-specific agonists (Pam3CSK4, Mfa1) in an IRAK-1-dependent but NF-κB-independent manner. These studies provide some of the first information to explain, mechanistically, how tobacco smoke changes the *P. gingivalis* phenotype in a manner likely to promote *P. gingivalis* colonization and infection while simultaneously reducing the host response to this major mucosal pathogen.

## Results

### Tobacco smoke exposure alters expression of *P. gingivalis* fimbriae and capsule

**Figure 1:**
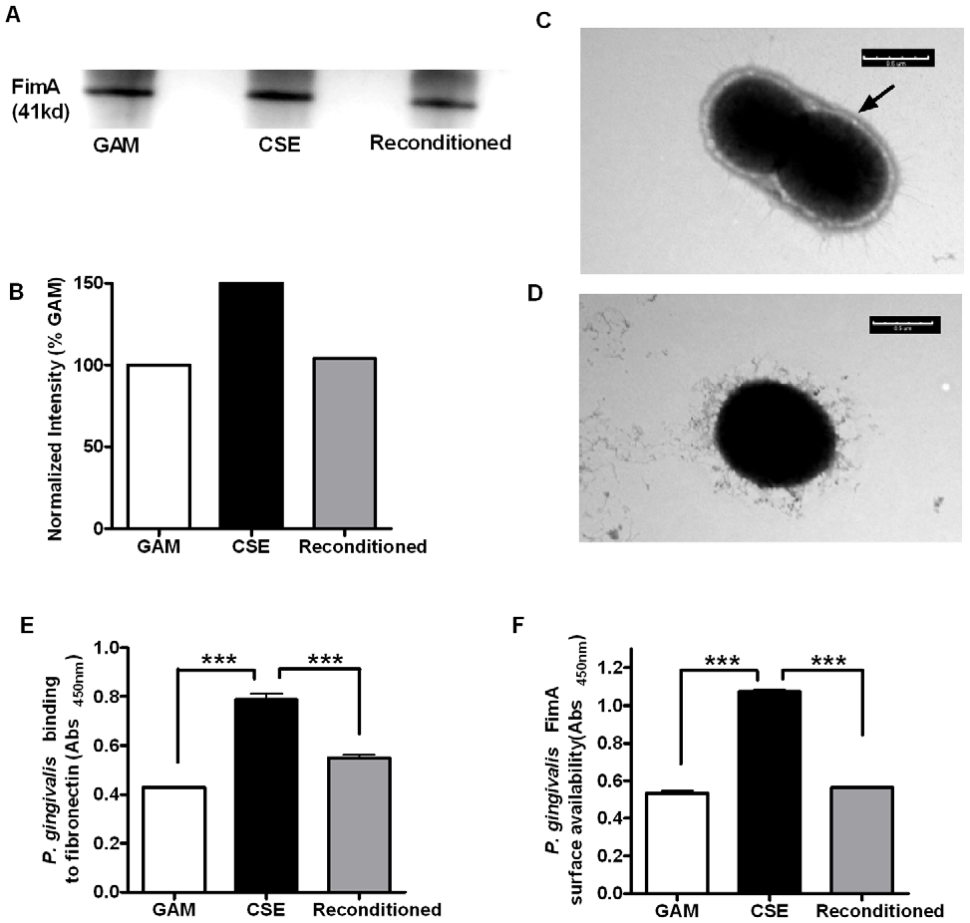
CSE induces phenotypic surface changes in *P. gingivalis.* (A) Typical Western blot of FimA in lysates of 1×10^6^ *P. gingivalis* cells sequentially passaged in GAM, GAM-CSE, and then fresh GAM, respectively. (B) Typical relative band intensities establish that FimA expression is increased on CSE-exposure, but that FimA expression reverts to control levels upon sub-culturing *P. gingivalis* back into fresh GAM. Representative transmission electron images of *P. gingivalis* grown in GAM (C) or GAM-CSE (D). The black arrow indicates the *P. gingivalis* capsule, which is greatly reduced in presence of CSE. These CSE-induced phenotypic changes are concomitant with an increased binding of *P. gingivalis* to the FimA ligand, fibronectin (E) and surface availability of FimA (F).

Our previous microarray data indicated that CSE induced the expression of genes key to the synthesis and assembly of FimA (PG2133 and PG2134), concomitant with a suppression of several genes in capsular biosynthesis locus (*capK*, PG0117, PG0118 and *wecC*) [14]. We now show that CSE-exposure reversibly increases FimA protein, as shown in ***Figures 1A and B***. Transmission electron micrographs clearly establish that these CSE-regulated transcriptional and translational activities are reflected at the ultrastructural level, as shown in ***Figures 1C and 1D***. Furthermore, CSE-induced FimA is both surface exposed, as assessed by availability to FimA-specific antibodies (***Figure 1F***), and functional, as assessed by binding to the established FimA ligand, fibronectin [24] (***Figure 1E***).

### CSE augments *P. gingivalis* biofilm formation

**Figure 2:**
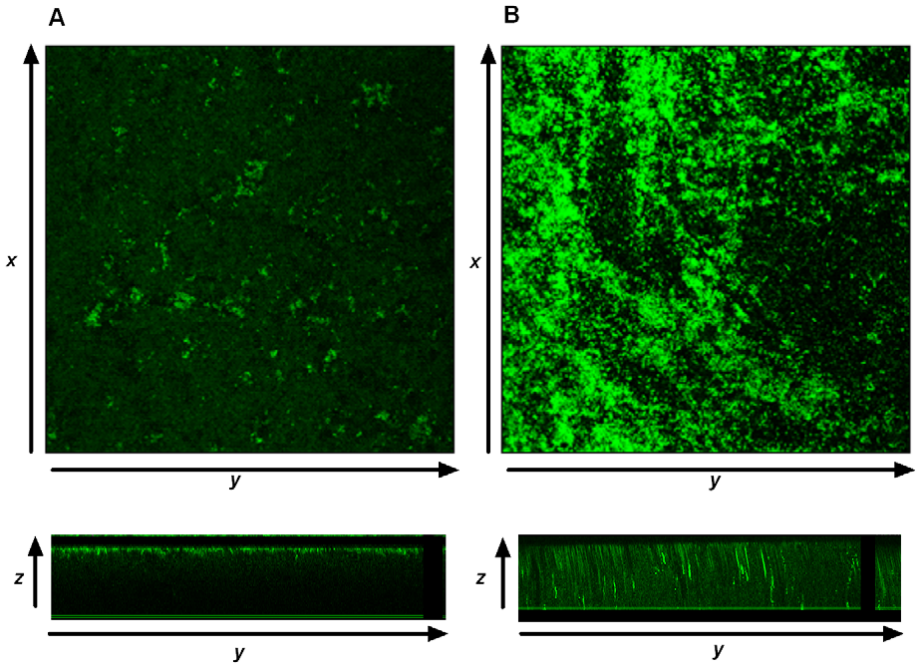
Visualization of homotypic biofilm formation by *P. gingivalis* grown in GAM or CSE. 48 hr *P. gingivalis* biofilms, formed in an open flow system, were stained with FITC. Optical sections were obtained along the *x-y* and *z* axis at 1 µm intervals. (A) and (B) are representative images of biofilms formed in GAM or GAM-CSE, respectively. Top panels represent *x-y* images while the bottom panel shows *x-z* stacks, reflecting biofilm thickness formed in GAM or GAM-CSE, respectively. *Z*-stack images were obtained from 5 randomly selected fields per biofilm and image data recorded along the *x-y-z* planes.

Biofilms play an important role in the pathology of periodontal disease by mechanisms that include protection of plaque bacteria against phagocytosis and against antibiotics [25]. FimA plays a critical role in *P. gingivalis* biofilm formation. Indeed, *P. gingivalis* strains lacking FimA cannot form biofilms [26]. Furthermore, capsule synthesis has been shown to inversely correlate with biofilm growth [16]. To establish if our initial discoveries - that CSE upregulates FimA production while downregulating capsule production [14] - influence biofilm formation, CSE-exposed and unexposed *P. gingivalis* was grown in an open flow biofilm system. Representative biofilm images are presented in ***Figure 2A-B***. There was a significant increase in overall homotypic biofilm formation in the presence of CSE (***Table 1***), with increased biomass, substratum coverage, and maximum and mean thickness apparent (all ***p***<0.01).

**Table 1:** Quantitative characteristics of *P. gingivalis* biofilms.

48h Biofilm	Biomass (µm^3^/µm^2^)	Substratum coverage (µm^2^)	Average thickness (µm)
*P. gingivalis* GAM	1.869±1.15	0.315±0.11	2.505±0.443
*P. gingivalis* GAM-CSE	5.708±0.38***	0.679±0.06	12.66±1.487**

Composite image data were analyzed using Matlab softwares to obtain biomass, average thickness; and substratum coverage of 48 h homotypic *P. gingivalis* biofilms formed with GAM and GAM-CSE.

**p<0.01; ***p<0.001.

### FimA and Mfa1 exhibit differential pro-inflammatory potential

**Figure 3:**
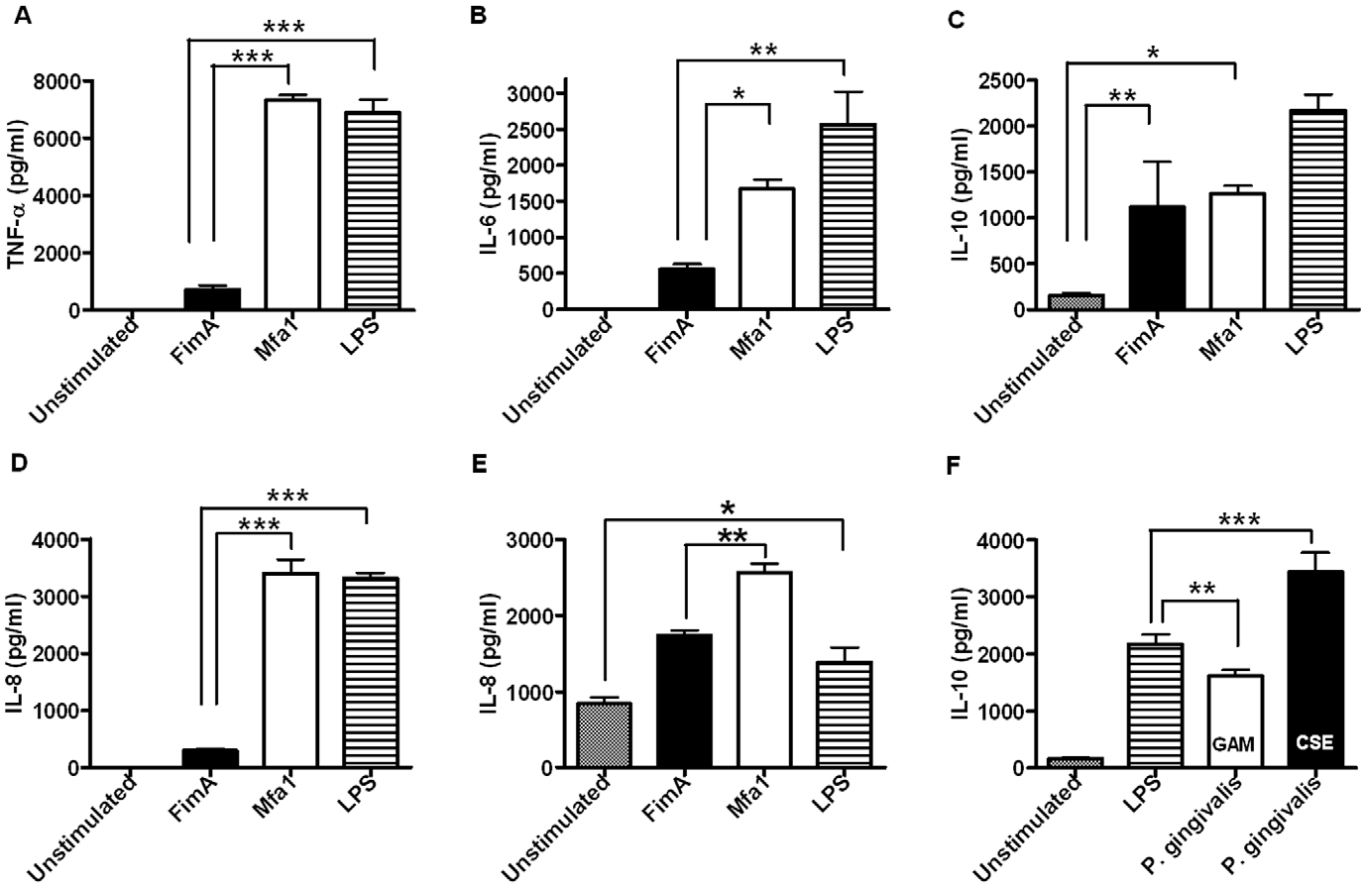
Inflammatory potential of rFimA and rMfa1. 0.5×10^6^ primary human PBMCs were stimulated with 1 µg/ml of rFimA, rMfa1 or the TLR2 and -4 specific agonists Pam3CSK4 and *E. coli* LPS, respectively. (A) TNF-α; (B) IL-6; and (C) IL-10 release was quantified by ELISA in 20 hr cell-free supernatants, harvested by centrifugation. (D) 0.5×10^6^ primary human neutrophils were stimulated with 1 µg/ml of rFimA, rMfa1, Pam3CSK4 or *E. coli* LPS. IL-8 release was quantified in 20 hr cell-free supernatants by ELISA.(E) Confluent OBA-9 epithelial cells (∼0.4×10^6^) were stimulated with 1 µg/ml of rFimA, rMfa1, Pam3CSK4 or *E. coli* LPS. IL-8 release was quantified in 20 hr cell-free supernatants by ELISA. (F) Similarly to purified FimA, whole, CSE-exposed *P. gingivalis* also induced increased IL-10 secretion from primary human PBMCs compared to control bacteria. *p<0.05; **p<0.01; ***p<0.001.

While the innate response to the major fimbrial antigen of *P. gingivalis*, FimA, has been partially characterized [27], the inflammatory potential of the minor fimbrial antigens are not well understood. Therefore, to better understand the relevance of CSE-upregulation of FimA and Mfa1, particularly in the context of the reduced inflammatory response to plaque that is consistently observed in human smokers [28], we quantified the cytokine production elicited by these predominant, CSE-regulated surface antigens in PBMC's (***Figure 3A-C***), neutrophils (***Figure 3D***) and gingival epithelial cells (***Figure 3E***). While FimA did promote a cytokine response from each cell type tested, the concentration of pro-inflammatory cytokines induced (TNF-α; IL-6; IL-8) was minimal when compared to the classic bacterial-derived pro-inflammatory agonists, LPS and Pam3CSK4. In contrast, FimA did induce high levels of the anti-inflammatory cytokine, IL-10 (***Figure 3C***). Mfa1, on the other hand, induced a robust pro-inflammatory response that was comparable or greater in magnitude to that of LPS in PBMC's (***Figures 3A and 3B***), neutrophils (***Figure 3D***) and epithelial cells (***Figure 3E***). These results are similar to those seen with intact bacteria. We have previously shown that whole, smoke-exposed *P. gingivalis* exhibit reduced inflammatory potential than control (unexposed) cells, as measured by decreased induction of multiple pro-inflammatory cytokines (TNF-α, IL-6 and IL-12 p40) [29]. We extend these observations to show that CSE-exposed *P. gingivalis* induces increased IL-10 secretion from innate cells, compared to unexposed bacteria (***Figure 3F***).

### FimA and Mfa1 are TLR2-specific agonists

**Figure 4:**
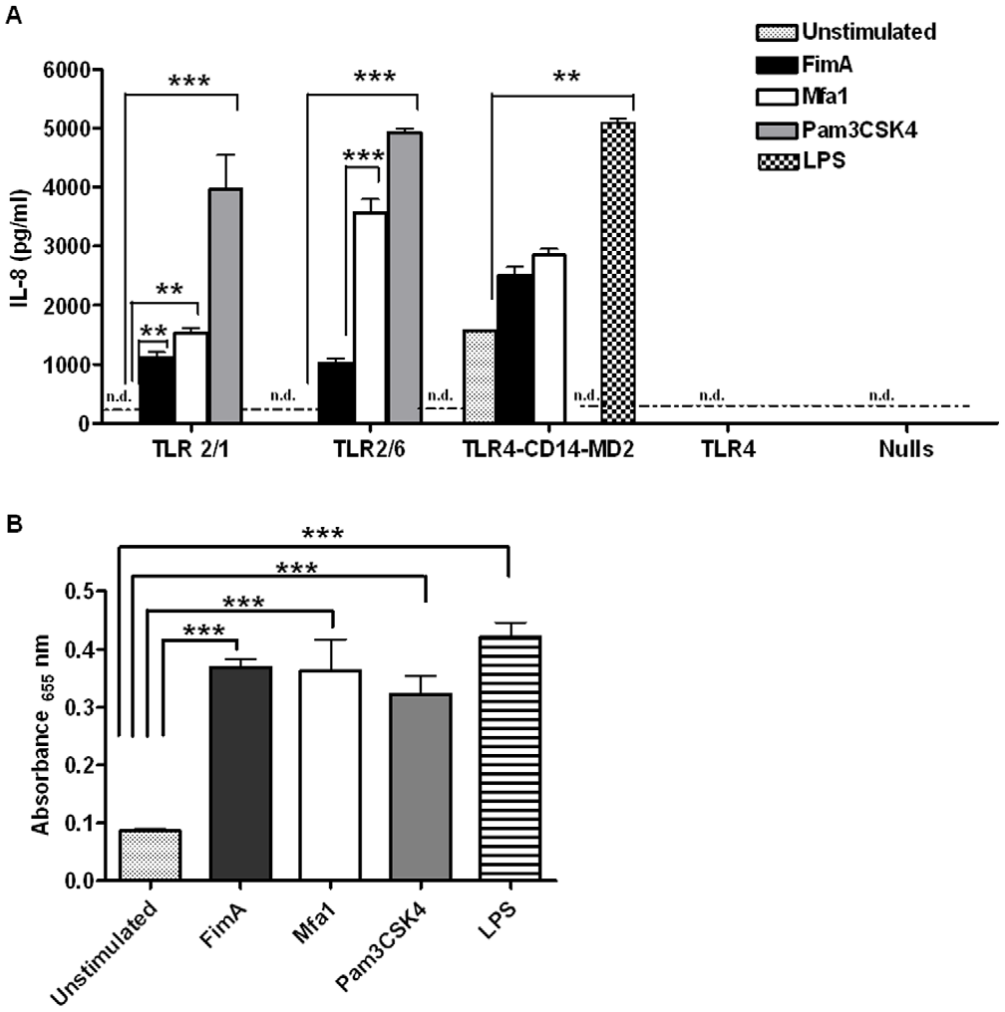
rFimA and rMfa1 signal preferentially through TLR2/6. (A) HEK 293 cells stably expressing TLR2, 4, 2/1, 2/6 or TLR4-CD14-MD2 were stimulated with 1 µg/ml of rFimA, rMfa1, the classic TLR2-specific agonist, Pam3CSK4 or the classic TLR4-specific agonist *E. coli* LPS. IL-8 release was quantified in 20 hr cell-free supernatants by ELISA. n.d.  =  not detected (below assay threshold); *p<0.05; **p<0.01; ***p<0.001. (B) THP-1 Blue cells are stably transfected with a reporter plasmid expressing secreted embryonic alkaline phosphatase (SEAP) gene under the control of a NF-κB-inducible promoter. THP-1 Blue cells were stimulated with 1 µg/ml of rFimA, rMfa1, Pam3CSK4 or *E. coli* LPS. Relative expression levels of SEAP (reflecting NF-κB) in cell-free supernatants were determined by spectrophotometric analysis of SEAP activity at 655 nm. Unstimulated cells represent the 100% control. Here we show that all TLR-agonists employed are equally capable of inducing NF-κB. ***p<0.001 compared to unstimulated cells.

In order to understand the differential pro-inflammatory potential of FimA and Mfa1 at the mechanistic level, we first established the TLR-specificity of each of these two CSE-dysregulated *P. gingivalis* surface proteins. IL-8 production by rFimA or rMfa1 stimulated HEK293 cells stably expressing variant TLRs was quantified. As shown in ***Figure 4A***, significantly higher levels of IL-8 were produced by HEK clones expressing TLR2/1 and, particularly, TLR2/6 compared to all other clones (both p>0.001).

As FimA induced lower levels of pro-inflammatory cytokines in innate immune cells than Mfa1, we hypothesized that FimA may not be a strong activator of NF-κB. However, in the THP-1 blue cell model, FimA and Mfa1 each proved to be effective inducers of NF-κB transcription (see ***Figure 4B***).

### FimA induces TLR2-specific innate tolerance

**Figure 5:**
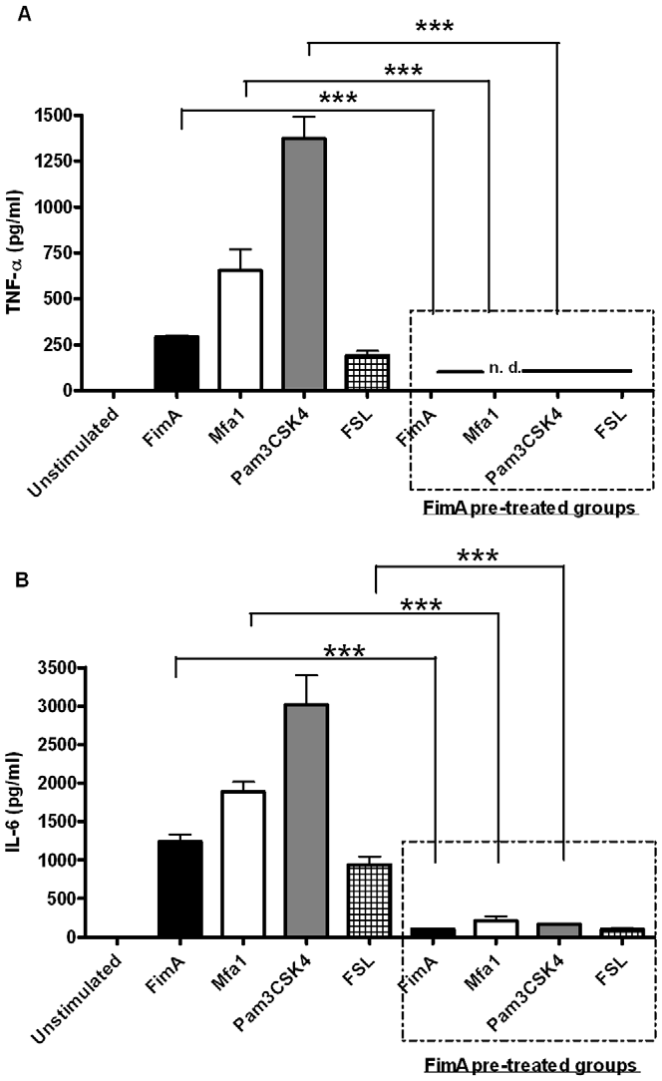
FimA induces TLR2-specific innate tolerance. 0.5×10^6^ primary human PBMCs were pre-incubated with rFimA for 24 hrs before stimulation with 1 µg/ml of rFimA, rMfa1 or the TLR2 and -4 specific agonists Pam3CSK4 and *E. coli* LPS. Responses were compared to cells treated with the same agonists without any pre-incubation. (A) TNF-α; (B) IL-6. n.d.  =  not detected (below assay threshold); ***p<0.001.

We next examined if exposure to low-activity FimA influenced cytokine production (IL-6, TNF-α) in PBMCs on subsequent TLR stimulation by potent pro-inflammatory agonists (Mfa1 and the TLR2/1 and TLR2/6 specific agonists, Pam3CSk4 and FSL respectively). Pre-incubation with FimA inhibited cytokine production in response to all TLR2 specific agonists tested, as shown in ***Figure 5***. Thus, FimA induces a state of inflammatory hypo-responsiveness in PBMCs. TLR2 surface expression by PBMCs in response to FimA stimulation was monitored by flow cytometry. There was no significant difference in TLR2 surface expression levels up to 24 hrs post-FimA engagement (*data not shown*). Thus, FimA stimulation does not alter surface expression of TLR2 in PBMCs.

### FimA stimulation does not induce IκBα degradation but inhibits IκBα degradation by Mfa1

**Figure 6:**
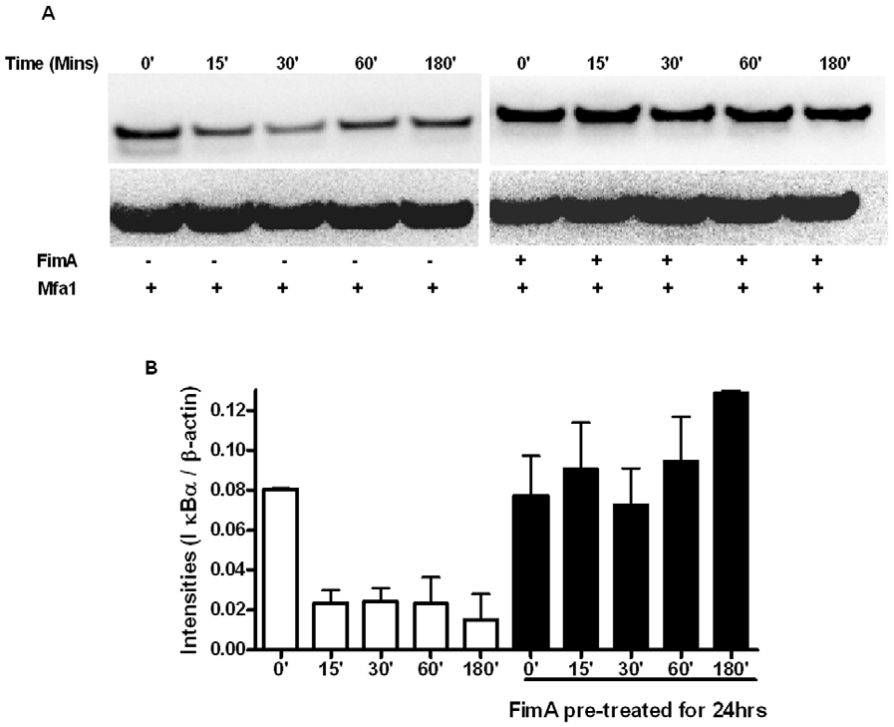
FimA induced tolerance reduces Iκ-Bα degradation. (A) Human PBMCs were pre-treated with 1 µg/ml rFimA for 24 hrs before stimulation with 1 µg/ml of rMfa1 for various timepoints. Immunoblots (25 µg protein per well) were probed for IκBα and re-probed β-actin to ensure equal loading. (B) Mean (s.e.) normalized band intensities.

NF-κB transcription factors are complexed with IκB proteins in the cytosol. On receiving specific, but varied, extracellular signals IκBα is phosphorylated and targeted for proteasome-mediated degradation, resulting in the release and nuclear translocation of transcriptionally effective NF-κB [9], [10], [30], [31], [32], [33]. We compared Iκ-Bα protein levels in PBMCs exposed to rMfa1, with or without pre-incubation with rFimA. As shown in ***Figure 6***, while the potent pro-inflammatory agonist, Mfa1, induced rapid and extensive Iκ-Bα degradation, pre-incubation with rFimA efficiently abrogated this Mfa1-triggered Iκ-Bα degradation. Thus, FimA appears to promote TLR2 hyposensitivity by inhibiting TLR2 agonist-induced degradation of Iκ-Bα. Essentially identical results were found for a second TLR2 agonist, FSL (*data not shown*).

### FimA induced innate tolerance is IRAK-1-mediated

**Figure 7:**
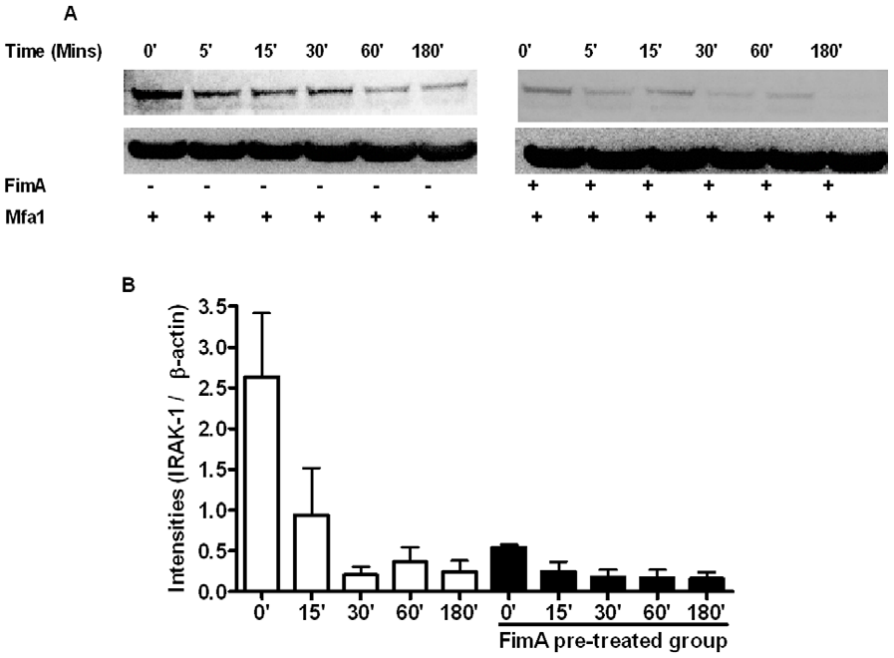
FimA induced tolerance is IRAK-1-mediated. Human PBMCs were pre-treated (B) or not (A) with 1 µg/ml rFimA for 24 hrs before stimulation with 1 µg/ml of rMfa1 for various timepoints. Immunoblots (30 µg protein per well) were probed for IRAK-1 and subsequently re-probed for IκBα and β-actin. (C) Mean (s.e.) normalized band intensities.

To further understand mechanisms underlying FimA-induced TLR hypo-responsiveness, we monitored cytosolic IRAK-M and IRAK-1 in PBMCs. In unstimulated PBMCs, exposure to TLR2 agonists resulted in the rapid (within minutes) degradation of the upstream NF-κB regulator, IRAK-1, as shown for Mfa1 in ***Figure 7A***. However, FimA stimulation not only degraded IRAK-1, without leading to induction of pro-inflammatory cytokine production (***Figure 3***) but IRAK-1 levels remained minimal 24 hours after FimA stimulation and, therefore, unavailable for signaling on secondary TLR2 stimulation, as is shown for Mfa1 in ***Figure 7B***. Essentially identical results were found for IRAK-M (*data not shown*).

## Discussion

Smokers are more susceptible to periodontitis and exhibit more severe disease, yet the normally overt inflammatory response to plaque bacteria is suppressed [14]. We have previously shown that human monocytes challenged with tobacco smoke-exposed *P. gingivalis* respond with reduced levels of pro-inflammatory cytokines [14] and now show that tobacco smoke-exposed *P. gingivalis* promote increased IL-10 production. However, the specific mechanisms of tobacco-induced innate immune suppression remain unknown. Initial microarray data suggested that cigarette smoke extract suppressed the production of *P. gingivalis* capsular polysaccharides while promoting the expression of fimbrial proteins [24], [34]. As both capsular polysaccharides and fimbrial proteins of this key periodontal pathogen have been reported to influence cytokine production and because these specific bacterial structures will be the first to engage the host response, we hypothesized that such smoke-induced changes to *P. gingivalis* surface may contribute to the lower pro-inflammatory potential of smoke-exposed bacteria.

We have herein established that tobacco smoke increases expression of FimA, the major fimbrial protein, reduces *P. gingivalis* capsular layer, and promotes the growth of *P. gingivalis* biofilms of increased biomass. FimA has been shown to play a critical role in *P. gingivalis* colonization of the periodontium through strong interactions with several host proteins, including collagen, laminin and fibronectin [35], and by promoting adherence to the oral epithelia [26] and to other plaque bacteria, such as *Streptococcus spp.* [36], [37]. Furthermore, it has been long established that biofilms represent immunoprotective structures. FimA is an important player in *P. gingivalis* biofilm formation [38]. Indeed, FimA deficient strains show reduced biofilm formation [16], while non-encapsulated mutants of *P. gingivalis* W83 exhibit enhanced biofilm formation [1], [39], [40]. These results are clearly in keeping with, and provide mechanisms to support, established clinical observations that show that, compared to non-smokers, smokers are more likely to be infected with *P. gingivalis* [41], [42], to harbor higher numbers of *P. gingivalis* [41], and for such *P. gingivalis* infections to be more persistent [43].

It has been previously shown that *P. gingivalis* FimA interacts with TLR2 [21], [23], [44], [45]. However, the capacity of FimA to subsequently elicit an inflammatory response is controversial. Some research groups have shown that FimA induces the production of pro-inflammatory cytokines from mononuclear innate cells [34], [45] and have hypothesized that this contributes to the development of pathological inflammation [21], [44], [46]. However, other studies suggest that the pro-inflammatory response to FimA in mononuclear innate cells may be significantly lower than that expected from comparable doses of classic TLR agonists [22] and is unlikely to play a major role in *P. gingivalis*-induced inflammation [47]. Moreover, it has even been suggested that FimA may be anti-inflammatory [28]. We now show that the CSE upregulated *P. gingivalis* fimbrial proteins, rFimA and rMfa1, each signal though TLR2/1, and particularly, TLR 2/6; both strongly activate NF-κB transcription; but that rFimA induces a minimal pro-inflammatory cytokine (TNF-α, IL-6) response in multiple innate cell types (human PBMCs, neutrophils and gingival epithelial cells) compared to rMfa1 and to classic TLR2 agonists; and also induces significant quantities of the potent anti-inflammatory cytokine, IL-10. These results mimic the cytokine profiles that we have previously shown in innate cells stimulated with intact, CSE-exposed *P. gingivalis* [9], [10], [48]. Furthermore, these observations are in keeping with multiple *in vivo* studies in smokers which have shown that, despite increased susceptibility to plaque-induced periodontitis, the inflammatory response is suppressed in smokers compared to non-smokers [49], [50], [51], [52]. This inflammatory suppression is reflected in lower concentrations of pro-inflammatory mediators (IL-1β, IL-6 and, perhaps, TNF-α) in the gingival crevicular fluid and periodontal tissues of smokers, compared to non-smokers [53], [54]. Inefficient TLR stimulation and the promotion of a less vigorous inflammatory response is an established means of immune evasion for several pathogens, including *Bordetella Shigella* and pathogenic *Escherichia* [55], [56], [57], [58].

While CSE induces functional, surface-exposed FimA expression, we further demonstrate in PBMCs that FimA induces a state of hyporesponsiveness, or tolerance, to secondary stimulation with Mfa1, and other TLR2 specific agonists - as demonstrated by an ablation of TNF-α and IL-6. This FimA-induced hyporesponsiveness was not associated with an alteration to TLR2 expression on the surface of innate cells. Rather, we show that FimA-induced, TLR2-specific tolerance is mediated by a suppression of Iκ-Bα degradation, which is expected to be concomitant with reduced Nf-κB translocation.

TLR2 activation leads to the rapid recruitment of MyD88 to TLR2 and the subsequent recruitment of IRAK-1 and IRAK-4 [59], [60]. IRAK-4 phosphorylates IRAK-1 that is in association with TLR2, allowing IRAK-1 to interact with TRAF6 [56]. This leads to IκBα degradation, NF-κB nuclear translocation and the induction of multiple pro-inflammatory cytokine genes [61]. We show, however, that stimulation with FimA leads to rapid IRAK-1 degradation and the long-term unavailability of this positive regulator of the TLR-2/MyD88 pathway, helping to explain FimA-induced tolerance. IRAK-M is a negative regulator of NF-κB translocation that acts by preventing the dissociation of IRAK-1 and IRAK4 from MyD88 [62]. While FimA stimulation also leads to the rapid degradation and chronic suppression of IRAK-M, this would appear inconsequential with respect to NF-κB activation because of the unavailability of IRAK-1.

This FimA-induced, IRAK-1- and IκBα-involved suppression of TLR2 pro-inflammatory cytokine signaling provides one mechanism by which tobacco smoke dysregulates the inflammatory response and suppresses cytokine production in smokers. This mechanism of suppression may be similar to what has been previously reported for Pam3CSK4 [62]. Monocytes stimulated with Pam3CSK4 have been shown to be refractory to secondary stimulation with the same agonist [28]. This occurs due to reduced IRAK-1 protein in these cells, just as we show in FimA-tolerized PBMCs.

It is important to note that there are limitations inherent in using recombinant proteins, particularly as they may behave differently outside the context of the whole bacterium and because of potential vector-specific alterations in the processing of the recombinant protein. The advantages, however, are that recombinant technology is long established and allows simple purification of a large amount of protein that can be readily characterized, is free of accessory molecules (such as associated fimbrial proteins FimB through E) and allows for effective and reproducible elucidation of signaling pathways. It is important to note that FimA and Mfa1 are simultaneously expressed [19], [38], raising the possibility that Mfa1 may nullify the anti-inflammatory effectiveness of FimA. However, FimA-comprised fimbriae are much longer appendages than the minor Mfa1 fimbriae [19], [38] and are, thus, likely to interact with host cells prior to Mfa1. Furthermore, it has been suggested that FimA appendages may be shed from the *P. gingivalis* surface [63] further increasing the likelihood of early FimA host interaction. Importantly, intact CSE-exposed *P. gingivalis* that, presumably, express both Mfa1 and FimA, exhibit similar anti-inflammatory properties to recombinant FimA. While tobacco smoke produces multiple phenotypic and genotypic changes in *P. gingivalis* [28] and, thus, other yet to be identified mechanisms may well contribute to the overall reduced inflammatory response, the anti-inflammatory effects of recombinant FimA are potent and are likely to be a major factor in CSE-induced inflammatory suppression.

In summary, CSE-exposure results in the upregulation of FimA. FimA suppresses the inflammatory response by at least two different mechanisms. One is passive, i.e. FimA itself engages TLR2 but has minimal inflammatory potential. The second is active, i.e., FimA induces TLR2 hyporesponsiveness by a mechanism involving IRAK-1 degradation and the stabilization of IκBα.

CSE mediated stress induced alterations in key virulence factors of *P. gingivalis*. While down-regulated capsular polysaccharides increased biofilm formation, upregulated fimbriae promoted TLR2 hyposensitivity in an IκBα-, IRAK-1- dependent manner. Both mechanisms, contribute towards altering host-pathogen interactions and reducing the host-induced inflammatory response. This is consistent with our prior observations that CSE-exposed whole P. *gingivalis* cells have reduced inflammatory potential compared to control bacteria [14]. These observations are also consistent with the clinical profiles of smokers of severe periodontal disease who lack overt signs of clinical inflammation [9], [10].

## Methods

### Reagents and antibodies

DMEM, RPMI 1640 and keratinocyte-serum-free (KSF) media, Dulbecco's PBS, bovine pituitary extract, Amphotericin B and Nu-Page 4–12% Bis-Tris gels and all other SDS-PAGE chemicals were obtained from Invitrogen (Carlsbad, CA). FBS came from HyClone (Logan, UT). THP-1, THP-1 Blue, TLR-transfected Human Embryonic Kidney (HEK) 293 and vector control cells as well as Pam3CSK4, FSL, LPS, normocin, blastocidin, zeocin, hygrogold and QUANTI-Blue NF-κB assays were purchased from Invivogen (San Diego, CA). Penicillin–streptomycin came from Mediatech (Manassas, VA). RIPA buffer, PhosSTOP and complete Mini EDTA-free protease inhibitor cocktail tablets were bought from Roche (Indianapolis, IN). BCA Protein Assay kits came from Pierce (Rockford, IL). SuperSignal West Pico Chemiluminescent Substrate kits and Bacterial Protein Extraction Reagent (B-PER) were purchased from Thermo Scientific (Rockford, IL). FimA-specific antibodies were custom generated by Cocalico Biologicals, Reamstown, PA, while FITC-labeled mouse anti- human CD16b and mouse IgM-FITC isotype control antibodies came from AbD Serotec (Oxford, UK). Fibronectin pre-coated microplates were from BD Biosciences (San Jose, CA). Anti-*P. gingivalis* antibodies were generated in house, while HRP-linked anti-rabbit IgG came from Sigma-Aldrich (St. Louis, MO). All other antibodies were purchased from Cell Signaling Technology (Beverly, MA) Gifu anaerobic medium (GAM) was bought from Nissui Pharmaceuticals (Tokyo, Japan). Lymphocyte separation medium (LSM) was purchased from MP Biologicals (Solon, OH). Dextran, PBS and tetramethylbenzidine were obtained from Fisher Scientific (Fair Lawn, NJ), EDTA from AA Hoefer Inc. (San Francisco, CA), while trypan blue, insulin, transferrin, 2-mercaptoethanol, 2-aminoethanol, sodium selenite, proteinase K acrylic beads and phosphotongstic acid were purchased from Sigma-Aldrich (St. Louis, MO). HiTrap Chelating HP affinity columns came from Amersham Biosciences Corp., (Piscataway, NJ). Isopropyl-β-D-thiogalactopyranoside (IPTG) was bought from RPI Corporation (Prospect, IL). Limulus amoebocyte lysates assay kit was purchased from Cape Cod Inc. (Falmouth, MA). IL-8 ELISA kits were from Cell Sciences (Canton, MA). TNF-α, IL-6 and IL-10 ELISA kits and FITC conjugated anti-human TLR2 and appropriate istoype control antibodies were purchased from eBioscience (San Diego, CA). Formavar coated copper grids were from Electon Microscopy Sciences (Hatfield, PA). Finally, standard reference cigarettes were obtained from Kentucky Tobacco Research and Development Center (Lexington, KY).

### *Porphyromonas gingivalis* growth, maintenance, and cigarette smoke extract (CSE) exposure

*Porphyromonas gingivalis* W83 and ATCC 33277 were purchased from the American Type Culture Collection (Manassas, VA) and maintained as frozen stocks. *P. gingivalis* was grown in GAM or GAM conditioned with CSE (GAM-CSE) under anaerobic conditions (80% N_2_, 10% H_2_, 10% CO_2_) at 37°C in a Coy Laboratories anaerobic chamber. Bacteria were harvested at mid- to late-exponential phase (O.D. of 1 corresponds to 10^9^ cells ml^−1^). GAM-CSE was prepared using standard 2R1 reference cigarettes, as we have recently described [64]. Briefly, cigarette smoke was drawn through 50 ml GAM by using a three-way stopcock and a syringe, with 35 ml ‘drags’ performed over a period of 2 sec, one drag every 20 sec. Cigarette smoke extract-conditioned medium was filtered (0.22 µm), and adjusted to pH 7.2. The nicotine content of GAM-CSE was determined by gas–liquid chromatography, as previously described [14] and GAM-CSE adjusted to physiologically relevant doses [14]. Unless otherwise noted, GAM-CSE was employed at a concentration of 4000 ng nicotine equivalents/ml. We have previously shown that *P. gingivalis* is tolerant, with respect to growth and viability, to such CSE doses [65].

### Human PBMC, neutrophil and gingival epithelial cell isolation and maintenance

Human peripheral blood mononuclear cells (PBMCs) were isolated from de-identified whole, citrated (10%) venous blood obtained from healthy donors by separation and collection of buffy coat and elimination of erythrocyte contamination with Histopaque 1077 density gradients, as previously described [66]. Written informed consent was obtained from all donors, and studies performed in compliance with University of Louisville, Institutional Review Board, Human Subjects Protection Program, (study number 503.05). Viability was routinely >98%, as determined by trypan blue exclusion. PBMCs were maintained in RPMI-1640 supplemented with 10% heat inactivated FBS, 100u/ml penicillin G and 100 µg/ml streptomycin, at 37°C, 5% CO_2_. Cells were allowed to rest overnight before addition of agonists.

Neutrophils were isolated from the erythrocyte-neutrophil pellet by collection from 1% Dextran- DPBS suspensions and elimination of erythrocyte contamination by hypotonic lysis. On restoring isotonicity, cells were washed once with cold DPBS and once with plasma buffer (2% plasma in DPBS). Neutrophils were suspended in RPMI-1640 supplemented with 10% heat inactivated FBS, 100 u/ml penicillin G and 100 µg/ml streptomycin, at 37°C, 5% CO_2_. Viability was routinely >98%, as determined by trypan blue exclusion. Purity of neutrophils was routinely ≥95%, as determined by flow cytometry using FITC-labeled anti-CD16b.

The human gingival epithelial cell line, OBA-9, was obtained from Dr. Denis Kinane (University of Pennsylvania, PA) and maintained at 37°C, 5% CO_2_ in KSF medium supplemented with 10 µg/ml of insulin, 5 µg/ml of transferrin, 10 µM 2-mercaptoethanol, 10 µM of 2-aminoethanol, 10 nM of sodium selenite, 50 µg/ml of bovine pituitary extract, 100 units/ml of penicillin/streptomycin and 50 ng/ml Amphotericin B.

### Analysis of capsule production and FimA expression on CSE-exposure

Mid- to late-log *P. gingivalis*, grown in GAM or GAM-CSE, were mounted on a formvar-coated copper grid, negatively stained with phosphotungstic acid at pH 7.0, and the bacteria visualized using a Philips CM-10 Transmission Electron Microscope. Total *P. gingivalis* lysate (1×10^5^ mid-late log cells) for Western blots were obtained from cells passaged twice in GAM, twice in GAM-CSE, or twice in GAM-CSE then reconditioned in fresh GAM. Western blots were probed with rabbit anti-FimA sera and HRP-linked anti-rabbit IgG. Immunoreactive bands were visualized by chemiluminescence. Imaging and densitometry were performed using the Kodak 4000MM Image station.

### Analysis of FimA binding activity and surface availability

Adhesion of mid- to late log phase CSE-exposed and control *P. gingivalis* cells to the extracellular matrix protein and FimA ligand, fibronectin, was measured by ELISA using fibronectin coated microplates, rabbit anti-*P. gingivalis* antibody followed by HRP-linked anti-rabbit IgG antibody and tetramethylbenzidine as the chromogenic substrate, essentially as previously described by Pierce et al [67]. Similarly, surface accessibility of FimA presented on mid- to late log phase CSE-exposed and control *P. gingivalis* cells was estimated by ELISA using rabbit anti-FimA antibody.

### Analysis of *P. gingivalis* biofilm formation

Formation of mono-species *P. gingivalis* biofilms was examined using a similar procedure to that described by Daep et al for bi-species biofilms [68]. Briefly, *P. gingivalis* cultures grown to late log phase either in GAM or GAM-CSE were introduced into BST FC 71 flow cells (Biosurface Technologies Corp., Bozeman, MT) with saliva coated cover slips at a flow rate of 6 ml/hr for 2 hr. Flow was maintained using a Manostat Carter 4/8 cassette peristaltic pump (Fisher Scientific, Suwanee, GA). Bacteria were then fed with either GAM or GAM-CSE for 48 hr. Resulting *P. gingivalis* biofilms were visualized by FITC staining. The depth of the FITC-labeled *P. gingivalis* biofilm was determined on an Olympus Fluoview confocal laser scanning microscope (Olympus, Pittsburgh, PA) from 5 frames randomly chosen by FluoView (Olympus, Pittsburgh, PA). Microcolony depth was determined by performing ***Z***-plane scans from 0 µm to 50 µm above the cover glass surface. Biofilm characteristics (depth, total surface area covered, substratum coverage, average and total thickness) were computed using Matlab v7.5.0 and Comstat (The Mathworks Inc., Natick, MA) [69].

### Purification of rFimA and rMfa1

Recombinant FimA and Mfa1 proteins were induced in *Escherichia coli* vectors containing FimA or Mfa1 cloned into pET-30 expression system, as described by Park et al. The C-terminal penta-histidine tagged recombinant proteins were induced in *Escherichia coli* by IPTG and purified using HiTrap chelating HP affinity columns. Purity of recombinant proteins was confirmed by SDS-PAGE. The lack of contamination by LPS, or other inflammatory mediators, was confirmed by several measures, that is, assessment of pro-inflammatory activity (TNF-α production by PBMCs) of recombinant proteins digested with proteinase K attached to acrylic beads; assessment of pro-inflammatory activity (TNF-α production by PBMCs) of recombinant proteins following boiling in 2% SDS; and the limulus amebocyte lysate assay.

### Pro-inflammatory profiling of FimA and Mfa1

PBMCs or neutrophils per well were seeded at 0.5×10^6^ in 96-well plates and stimulated with 1 µg/ml rFimA, rMfa1, or TLR-specific agonists (*E. coli* LPS, Pam3CSK4 and FSL). TNF-α, IL-6, and IL-10 (PBMC) and IL-8 (neutrophil) levels were determined in 24 hr supernatants by ELISA. OBA-9 cells were seeded at 0.1×10^6^ cells per well in 12-well plates. On reaching confluence (48 hr) the epithelial cells were stimulated with rFimA, rMfa1, LPS, Pam3CSK4 and FSL. Twenty-four hr OBA-9 culture supernatants were assayed for IL-8 by ELISA.

### TLR-specificity of FimA and Mfa1

Human Embryonic Kidney (HEK) 293 cells expressing either TLR2/1, TLR2/6, TLR4, TLR4-CD14-MD2 and HEK TLR null cells were maintained at 37°C in 5% CO_2_ in DMEM medium supplemented with 10% FBS, 100 µg/ml normocin and 10 µg/ml blastocidin. TLR4-CD14-MD2 cells were also provided 50 µg/ml hygrogold. TLR activation in each cell line was measured as IL-8 induction 24 hr post-stimulation with rFimA, rMfa1 or specific TLR positive control agonists.

### Induction of TLR2 tolerance by FimA

To determine if rFimA induced TLR2-specific tolerance, PBMCs (1×10^6^ cells per well) were stimulated, or not, with rFimA (1 µg/ml) in 5 ml polypropylene tubes. 24 hr culture supernatants were collected, PBMCs washed once with DPBS and re-suspended in RPMI. The cells were then restimulated with FimA, Mfa1, Pam3CSK4 or *E. coli* LPS (all 1 µg/ml) for an additional 24 hrs. TNF-α and IL-6 secretion was measured by ELISA.

### NF-κB induction by FimA and Mfa1

THP-1 Blue cells, maintained at 37°C, 5% CO_2_ in RPMI supplemented with heat inactivated FBS, 100 u/ml penicillin G and 100 µg/ml streptomycin and 200 µg/ml zeocin, were stimulated with FimA, Mfa1 or TLR agonists in 96 well plates (0.5×10^6^ cells per well) for 24 hrs. Nf-κB activation was quantified by using the QUANTI-Blue assay, according to the manufacturer's protocol.

### TLR2 surface expression following FimA stimulation

PBMCs (1×10^6^ cells) were treated with FimA (1 µg/ml) or left unstimulated for 24 hrs. Surface expression or redistribution of TLR2 was quantified by flow cytometry using FITC-conjugated anti-TLR2 and istoype control antibodies.

### Involvement of IRAK-1, IRAK-M in FimA-induced TLR2 tolerization

Whole cell lysates from 2.5 million PBMCs stimulated with 1 µg/ml FimA, and unstimulated controls, were prepared using RIPA lysis buffer with a phosphatase and protease inhibitor cocktail. Total protein was determined by BCA assay according to the manufacturer's protocol. Western blots (30 µg protein) were probed for IRAK-1, IRAK-M, Iκ-Bα and β-actin. Immunoreactive bands were visualized by chemiluminescence. Image analysis and densitometry were performed using the Kodak 4000MM Image Station. Bands intensities were normalized to the loading control (β-actin) and expressed as relative intensities.

### Statistical analysis

All experiments were carried out a minimum of three times, unless otherwise noted. Statistical significance between groups was evaluated by one-way nonparametric ANOVA and the Tukey multiple-comparison test using the InStat program (Graph-Pad Software, San Diego, CA). Differences between groups were considered significant at the level of *P*<0.05.
